# The Efficacy Study on Si Ni San Freeze-Dried Powder on Sleep Phase in Insomniac and Normal Rats

**DOI:** 10.1155/2013/947075

**Published:** 2013-05-30

**Authors:** Yuefeng Li, Angguo Liu, Ying Wang, Xingke Yan

**Affiliations:** ^1^Department of Pharmacology of Chinese Materia Medica, Gansu University of Traditional Chinese Medicine, Lanzhou, Gansu 730000, China; ^2^Department of Pharmacology, Dali University, Dali, Yunnan 671000, China

## Abstract

*Objectives*. To investigate the effect of Si Ni San freeze-dried powder (SNSP) on sleep phase in insomniac and normal rats, to identify its mode of action in improving sleep, and to provide a reliable method for determining pharmacodynamic material basis of Si-Ni-San on improving sleep. *Methods*. Rats were deprived of sleep by using the footplate electrical stimulator to record the rats' electroencephalogram (EEG) and electromyogram (EMG) by using polysomnography (PSG) and copy insomnia model by the method of electric stimulation. Analysis on EEG and EMG was carried out to observe the effects of SNSP on the sleep phase of insomniac and normal rats. *Results*. Rats were treated by intragastric administration (i.g.) consecutively for seven days. The results showed that the total sleep time was extended; meanwhile, SWS2 (*P* < 0.01) and REMS (*P* < 0.05) were mainly prolonged for both insomniac and normal rats. The dates implied SNSP could significantly improve sleep. *Conclusions*. SNSP could prolong SWS2 and REMS, the experimental reproducibility is good, and the dates indicate that SNSP has the sedative function. Study on the effects of SNSP on sleep phases provided a basis for the further studies on effective constituents and the pharmacodynamic mechanism of SNSP.

## 1. Introduction

With the accelerated pace of modern social life and work pressure, improving sleep quality has increasingly become the focus of attention. Sleep disturbance is a common disease and a variety of diseases' associated symptom. As necessary as sleep is for life, it is an important part of the body recovery, integration and consolidation of memories, indispensable part of health. 

Sedative and hypnotic chemical drugs mainly decrease slow-wave sleep 2 (SWS2) and rapid-eye-movement sleep (REMS) and relatively increase slow-wave sleep 1 (SWS1) to prolong the total sleep time (TST). After repeated administration, addiction and dependence will appear. Moreover, many adverse effects such as abstinence symptoms and rebound phenomena will appear following drug withdrawal [1]. Traditional Chinese medicines have important effects in treating insomnia and have fewer side effects compared with chemical drugs. Si Ni San freeze-dry powder (SNSP) is considered a typical medicine.

Si Ni San was first recorded in Shang Han Lun by Zhang Zhongjing in Eastern Han Dynasty, and it consisted of four herbs that are bupleurum, white peony, immature bitter orange, and licorice. It is the basic prescriptions of traditional Chinese medicine (TCM) to alleviate Shaoyang and coordinate liver and spleen. Si Ni San can disperse pathogens, alleviate mental depression, sooth the liver, regulate the spleen, and exhaust the stagnation of qi and blood. In recent years, studies found that SNSP has a unique curative effect in sedative-hypnotic and raising the quality of sleep, after the experiment of sedative hypnotic pharmacodynamics of SNSP [2–5]. According to clinical data of traditional Chinese medicine, insomnia induced by stagnation of Liver-qi accounts for more than 80% of all insomnia patients. Therefore, we select a representative prescription Si Ni San with the function of relieving the depressed liver and harmonizing liver and spleen as the research objective and discuss its pharmacological effects on improving sleep. Compared with chemical drugs, Si Ni San has important effects in treating insomnia and has fewer side effects. Efficacy study on Si Ni San of sleeping time of rats is carried out, which will provide a basic data for developing new traditional Chinese medicine drugs to improve sleep with high performance and low toxicity and provide information for the further studies on effective constituents and the effecting mechanism of Si Ni San.

The aim of this study is to verify the effect of SNSP on sleeping states of rats by recording the cortical electroencephalography (EEG) of insomniac rats and normal rats and calculating the total duration of each sleeping state, which will provide a reliable method for determining the pharmacodynamic material basis of Si Ni San on improving sleep. 

## 2. Materials and Methods

### 2.1. Animals

This experiment was performed in the Department of Pharmacology of Chinese Materia Medica, Gansu University of Traditional Chinese Medicine from January 2008 to July 2011.

Male Wistar rats, weighing 220 ± 10 g, were purchased from the Experimental Animal Centre of Gansu University of Traditional Chinese Medicine. Before the study, the animals were acclimated for 5 to 7 days in temperature (20–22°C) and humidity (40–45%) controlled rooms with a 12 h light cycle. All experiments followed a protocol approved by the local Animal Ethics Committee and the local government.

### 2.2. Instruments

There are 16-channel physiological signal recorder (Stoelting Company, USA); electromagnetically shielded recording chamber; stereotaxic apparatus (Stoelting, USA); plexiglass boxes; footplate electrical stimulator (Lanzhou Research Institute of Electrical Instruments). 

### 2.3. Reagents and Chemicals

Pentobarbital sodium (batch no. 080605) was purchased from Shanghai General Reagent Factory, Shanghai, China. It was prepared with distilled water to a 1% solution before use. Dental acrylic water (08-05-07) and dental acrylic cement (batch no. 09-07-02) were purchased from Gansu Dental Equipment Factory and benzylpenicillin sodium (batch no. A09078537) from the General Pharmaceutical Factory of the Gansu Pharmaceutical Group. Components of SNS, Bupleurum root, radix Paeoniae alba, bitter orange, and licorice were kindly authenticated by Dr. Chengyi Li, Professor of Pharmacognosy. 

### 2.4. Methods

#### 2.4.1. Preparation of Si Ni San Freeze-Dried Powder (SNSP)

**Figure 1:**
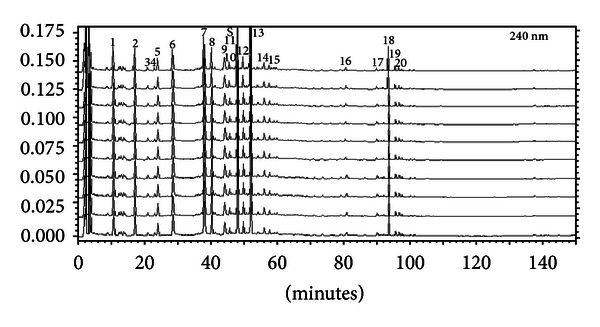
HPLC-FP of Si Ni San (freeze-dried powder).

**Figure 2:**
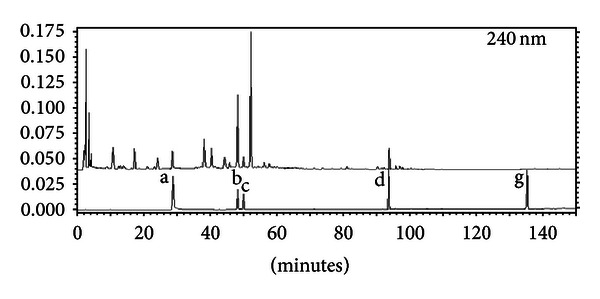
Contrast analysis of the HPLC chromatogram of Si Ni San (freeze-dried powder) and mixed standard substance a: peoniflorin, b: naringin, c: aurantiamarin, d: ammonium glycyrrhizinate salt, e: saikoside A, f: saikoside D, and g: enoxolone.

The mixture (580 g) of Bupleurum root, Radix Paeoniae Alba, bitter orange, and licorice was decocted for 30 min with boiling distilled water (equal to 10-fold the weight of the mixture) and then filtered. The drug residue was decocted for 20 min with boiling distilled water (equal to 6-fold the weight of the mixture) and then filtered. Filtrates from the two decoctions were put together, concentrated to the required volume, and prepared by freeze-drying processed Si Ni San freeze-dried powder. The constituents in Si Ni San freeze-dried powder were detected, and the four major ones of them (including paeoniflorin, naringin, hesperidin, and licorice acid) were quantified by HPLC (Figures 1 and 2, Table 1).

#### 2.4.2. Rat Sleep Phase Distinguishing Standard and Quantitative Analysis Index

**Figure 3:**
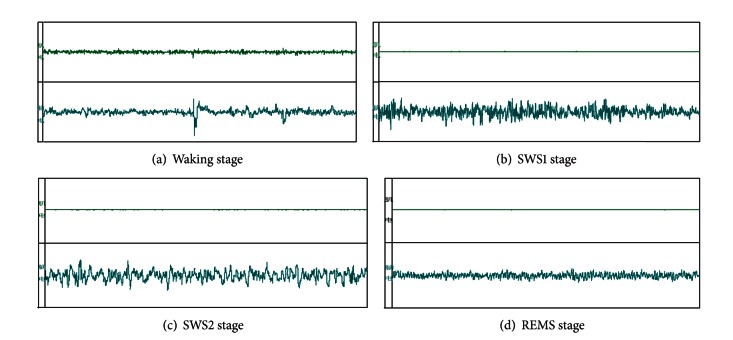
EMG and EEG of waking and sleep phase in freely moving rats.

Each 30 s is a segmented time, and the sleep-wake cycle of rats can be divided into four states [6–12] (Table 2) based on the wave form (Figure 3).

#### 2.4.3. Implantation for EEG

After the animals were anesthetized with pentobarbital sodium (45 mg/kg) and fixed in the stereotaxic apparatus, the skull was exposed. Two screw electrodes (1 mm diameter) were implanted into the skull (AP-2, R2; AP+2, R2) as cortical electrodes, and another one was placed at the center of the frontal bone (AP+5, R0) as a ground electrode. The cortical electrodes must touch the dura, but not cut it. The electrodes were connected to a socket by leads, which were fixed on the skull with dental acrylic cement [13–16]. Postoperatively, rats were put into separate plexiglass boxes and housed in an electromagnetically shielded recording chamber under standard conditions of temperature (21 ± 2°C), humidity (40%–45%), lighting (7:00–21:00 h), and ventilation. Each rat was administered intraperitoneally with 45 000 U penicillin for three days and allowed to recover for seven days. Before testing, the EEG recording cable was connected to a socket for 5.5 h for habituation to the experimental conditions. During EEG recording, the behavior of the rats was observed using a video monitoring system.

#### 2.4.4. Replication of Insomniac Model in Rats

On the 8th day after operation, the EEG signals of rats in a nonstressed state were recorded. Recording time lasted for 10 h from 08:00 to 18:00 h. The next day, animals were placed in separate plexiglass boxes (14 mm × 25 mm × 28 cm), on an electrified grid, through which electric shocks were delivered. The shock intensity was 0.5 mA, 1 Hz, and 18 ms long [7]. The electric shocks lasted for 30 s with a 30 min interval between sessions. At the same time, the EEG signals were recorded. After the test, the EEG and EMG were analyzed by the outcome measures of W, SWS1, SWS2, and REMS using the paired Student's *t*-test to check if the insomniac models were replicated successfully.

#### 2.4.5. The Effect of Si Ni San Freeze-Dried Powder on Sleep Phase in Normal Rats

The animals were placed in a quiet laboratory, and the recording time lasted for 4.5 h from 09:30 to 14:00 h. Rats were housed under standard conditions of humidity (40%) and temperature (20°C). Rats' embedded recording electrodes were randomized into two groups, with fifteen rats in each group. Rats were ensured light and dark alternately for 12 h every day and allowed to recover for two days after operation. Rats were administrated with SNSP 5.0 g/kg (containing about paeoniflorin 43 mg, naringin 246 mg, hesperidin 19 mg, and licorice acid 20 mg) for seven consecutive days. Thirty min after the last administration, the EEG recording cable was connected to sixteen physiological signal recorders by a socket and a connecting line, and the sampling rate was 500.000 samples/sec. EEG and EMG signals of each rat were recorded for two days, and recording results were used averagely. 

#### 2.4.6. The Effect of Si Ni San Freeze-Dry Powder on Sleep Phase in Insomniac Rats

Rats' EEG and EMG signals were recorded in a nonstressed state on the 8th day after operation, recording under nonstress state of normal rat electroencephalogram (EEG) and electromyogram (EMG) signals. The tracing time was 9:30–17:30 for total 8 h. Nine am the next day, the same rats were given the same volume of distilled water by gavage again, and then the rats were placed in a cage electric gate at the bottom of insomnia stimulator; the rats' feet were bottom stimulated for 8 h. The impact strength was 0.5 mA, 15 ms wide, and 1 Hz, during stimulus EEG, and EMG recording line can automatically interrupt it. Electrical stimulation can be started by two adjustable timers automatically. Thus, each stimulus was 30 s with intervals of 30 min to cause rats' insomnia. Tracing time was from 9:30 to 17:30 after the end of the stimulation. EEG and EMG were analyzed after the test. Animals with the reduced SWS1, SWS2, and TST were used as the experimental mice. After three days, insomnia rats were administrated with SNSP 5.0 g/kg (containing about paeoniflorin 43 mg, naringin 246 mg, hesperidin 19 mg, and licorice acid 20 mg) for seven consecutive days. Thirty min after the last administration, the EEG and EMG signals of each rat in an electrically stimulated state were recorded for 8 h from 09:30 to 17:30 h. The variation characteristics of EEG were analyzed for pre- and poststimulation and different periods of administration.

#### 2.4.7. Statistical Analysis

The first author analyzed the raw data statistically. All the results are analyzed with SPSS 17.0 using the paired Student's *t*-test.

## 3. Results

### 3.1. Analytical Results of the Effect of Si Ni San Freeze-Dry Powder on Sleep Phase in Normal Rats (Figure 4, Table 3)

**Figure 4:**
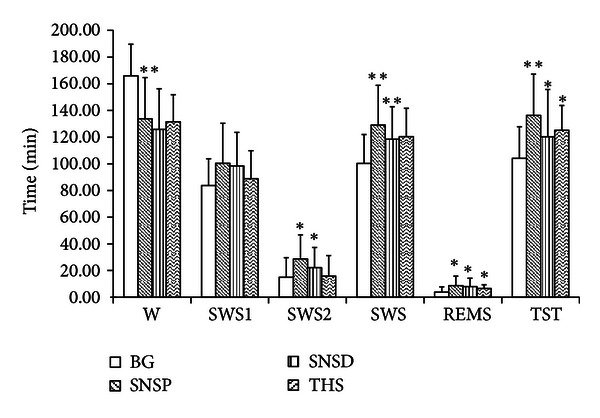
The effect of Si Ni San freeze-dry powder on sleep phase in normal rats.

Results in Figure 4 and Table 3 were shown after rats were administrated with SNSP 5.0 g/kg (containing about paeoniflorin 43 mg, naringin 246 mg, hesperidin 19 mg, and licorice acid 20 mg) for seven consecutive days. The time of the awakening of the normal rats' decreased significantly (*P* < 0.01), TST increased markedly (*P* < 0.01), SWS2 (*P* < 0.05), REMS (*P* < 0.05), and SWS increased, and SWS1 was not obviously affected. After the rats were administrated with SNSP for seven consecutive days, the effects of SNSP on prolonging the total sleeping time of mice were significantly stronger than those in the control group, and the effects of SNSP on prolonging the total sleeping time of mice were significantly stronger than those In the Si Ni San decoction (SNSD) and tui hei su (THS) groups.

### 3.2. Analytical Results of the Effect of Si Ni San Freeze-Dry Powder on Sleep Phase in Insomnia Rats (Figure 3, Table 2)

**Figure 5:**
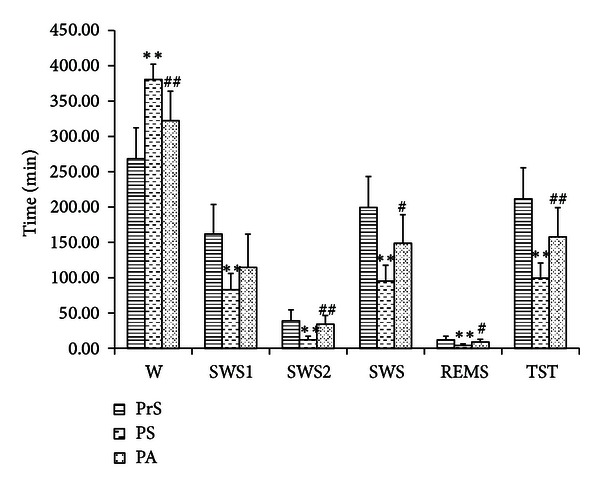
The effect of Si Ni San freeze-dry powder on sleep phase in insomniac rats.

Result in Figure 5 and Table 4 showed that significant differences were found for W, REMS SWS1, SWS2, and TST by comparing pre- with postshock (PrS and PS), indicating that the model was successful in replicating insomnia. The time of the awakening of insomniac rats decreased significantly (*P* < 0.01), but TST was significantly longer than preadministration with Si Ni San freeze-dried powder (*P* < 0.01), SWS2 increased markedly (*P* < 0.01), REMS increased significantly (*P* < 0.05), and SWS1 was not significantly longer than preadministration.

## 4. Discussion

So far, efficacy study on Si Ni San on sleeping time is limited to the clinical observation phase. In this study, research methods on the central nervous system pharmacology are used, and efficacy study on Si Ni San on sleeping time induced by pentobarbital sodium is carried out on mice, which elucidates its pharmacological effects on improving sleep.

The present study aimed to investigate the effect of SNSP at pre- and postshock (PrS and PS) on states of the sleep phase in insomniac Wistar rats using a modern experimental model. The effects of SNSP on prolonging the total sleeping time of rats were significantly stronger than those in the control group. The results showed that Si Ni San can increase markedly the sleeping time and also has a distinct pharmacological action in ameliorating insomnia as compared to synthetic drugs, because Si Ni San acts by extending SWS2 and REMS to increase the total sleeping time, and the effects of SNSP on prolonging the total sleeping time of mice were significantly stronger than those in the Si Ni San decoction (SNSD) and tui hei su (THS) groups. Si Ni San is likely to cause few side effects, and SNSP and SNSD could prolong the mouse sleeping time (*P* < 0.01), especially SNSP (*P* < 0.01), proving that SNSP has a very significant effect on insomnia treatments.

SNSP showed an internal role in improving sleep, which has been found in the cerebrospinal fluid component analysis studies. This explains that SNSP has some unique pharmacological effects on improving sleep role and has great advantages compared to synthetic drugs [1].

This study establishes the basis for clinical use of SNS and provides experimental evidence underlying its mechanism of action.

## Figures and Tables

**Table 1:** The contents of five marker constituents in Si-Ni-San (freeze-dried powder) determined by 3D-HPLC.

Compound	Paeoniflorin	Naringin	Hesperidin	Licorice acid
Contents^a^ (mg/g)	507.4 ± 0.03	167.5 ± 0.01	135.1 ± 0.02	136.6 ± 0.02

^a^Data expressed as mean ± SD (*n* = 4).

**Table 2:** Sleeping states differentiation.

(a) Waking (W)	During W, EEG signals are different when rats are in different behavioral conditions, and there are two kinds of EEG signals: when rats are moving, climbing, exploring, or scanning, cortical EEG waves are predominantly theta rhythm (6–9 Hz) waves, and when rats are grooming or standing still, cortical EEG waves are predominantly low voltage waves with high frequency.

(b) SWS1 SWS2	When rats are lying, eyes closed, or sleeping, cortical EEG waves are predominantly high amplitude waves (0.5–5 Hz) with sleep spindles (10–15 Hz). During SWS1, high amplitude waves occupied less than 50% of the period. SWS2 is characterized by high amplitude waves with low frequency and also sleep spindles. High amplitude and low frequency waves occupied more than 50% of the period.

(c) Rapid eye-movement sleep (REMS)	REMS is characterized by theta waves which are not markedly different from W. Thus, REMS is determined according to the EEG signal together with the behavior of the rats. Because waking cannot transform into REMS directly, SWS must appear before REMS, while REMS can return to SWS or W directly. Generally, the duration of REMS is less than 3 min. Any separate state lasts for at least 20 s, and a period of 20 s is considered an analytic unit.

(d) TST	Total sleep time (TST) includes the SWS1, SWS2, and REMS.

**Table 3:** The effect of Si-Ni-San freeze-dry powder on sleep phase in normal rats ($\overset{-}{x}$  ± SD).

	*n*	W	SWS1	SWS2	SWS	REMS	TST (min)
BG	15	165.93 ± 23.75	83.77 ± 20.04	14.97 ± 14.61	100.27 ± 21.68	3.80 ± 3.95	104.07 ± 23.75
SNSP	15	133.70 ± 30.95**	100.40 ± 29.97	28.67 ± 18.06*	129.07 ± 29.82**	8.73 ± 7.12*	136.30 ± 30.95**
SNSD	15	125.83 ± 30.42	98.33 ±25.21	22.17 ± 15.09*	118.51 ± 24.33**	7.89 ± 6.35*	120.26 ± 35.47*
THS	15	131.52 ± 20.33	88.84 ± 21.03	15.85 ± 15.32	120.35 ± 21.25	6.55 ± 2.78*	125.11 ± 18.58*

**P < 0.01, *P < 0.05 versus BG: blank group; SNS: Si-Ni-San.

THS (melatonin) tui hei su (melatonin is a natural hypnotic substance that is deep within the brain-like echinacea size “the pineal gland secretion of an amine hormone, so some people call it the pineal gland”); SNSD Si-Ni-San decoction (SNSD: is hypnotic-sedative herbs).

**Table 4:** The effect of Si-Ni-San freeze-dry powder on sleep phase in insomnic rats ($\overset{-}{x}$  ± SD).

	*n*	W	SWS1	SWS2	SWS	REMS	TST (min)
PrS	8	268.31 ± 44.02	161.88 ± 41.80	38.94 ± 15.63	199.56 ± 43.56	12.13 ± 5.13	211.69 ± 44.0
PS	8	380.63 ± 21.52**	83.13 ± 22.80**	12.06 ± 5.22**	95.19 ± 22.39**	4.19 ± 2.19**	99.38 ± 21.52**
PA	8	322.25 ± 41.61^##^	114.69 ± 46.97	34.13 ± 12.41^##^	148.81 ± 40.47^#^	8.94 ± 3.70^#^	157.75 ± 41.61^##^

**P < 0.01, *P < 0.05, versus PrS; ^##^ *P* < 0.01, ^#^ *P* < 0.05, versus PrS: pre-shock, PS: post-shock, PA: post-administration.

## Abbreviations

SNSP: Si Ni San freeze-dried powder
SNSD: Si Ni San decoction
EEG: Electroencephalogram
EMG: Electromyogram
PSG: Polysomnography
SWS1: Slow-wave sleep 1
SWS2: Slow-wave sleep 2
TST: Total sleep time
REMS: Rapid-eye-movement sleep
TCM: Traditional Chinese medicine
THS: (Melatonin) tui hei su
BG: Blank group
PrS: Preshock
PS: Postshock
PA: Postadministration.
